# Supplementary material for: Chromothripsis during telomere crisis is independent of NHEJ, and consistent with a replicative origin
Source: Genome Res. 2019 May;29(5):737–49. doi: 10.1101/gr.240705.118 (PMC6499312; doi:10.1101/gr.240705.118)
Supplement: Supplemental Material [file supp_gr.240705.118_Supplemental_file_1.zip › contigs/annotated_contigs/DB116/contig.2.DB116_length_758_mean_cov_22.1372031662.docx]

**DB116_length_758_mean_cov_22.1372031662**

```
CTTTTTACAAATCTCTTAACTTCCTTAAACGTTGTGCTTGTTTGCGGTTCTCCCAATGATGATCACATCATTTCCTGAACATTCCCAGG
 >chr10:20648662-20649012 + E=7e-185
GTGAACTCAATGCAGATTCTTCAGATTTTAGGGAAAACGTGGATTGGTAGAAAAAAAACAAAAATGACCATGGTTTTCAAAATTTTTTT

TCTATATTCTTCCCCATTGACATACTGTACAGTTTAAAAGTGTCCTATTATACTATATACTGAGATATTAAGCATAAAATCTTTCCTTA

GATATGTTCCTATGACATTTCATCCATTTCCAATAAAACATGTGGGTAAGTAAGATGTAGTGGATGTCAAAACTAATAGGTT|ATT|AC
                                                                                      >
ATTCGTAATAATCTCCTTTTGAATCTTGATGTTTTCTGTAACCCTGGTAGCTAAGTGCTGATAACAACAATTATCTAAATGTTGCAAAA
chr10:21386903-21387309 + E=2e-229
GGATGAAATCAGTAACAATGGGTCTACTGAAAAGAGGCAAAAATGTGAGTGGATATGAATTTATAACAGCAATAGGGCTAAAGCTGCAG

GGCCCTTTCATGTAGGAGGATAGCAACACACACACACACTCATTCACATACACACACATTCTCACTCACACGTGCACACATACGTACAC

ACACTTGTACACATTCACACACACTGTCACACAGAGAGTGCAGAAATGAAAGGAGACATTATCACGCATCCACACAAAAGACAAAGATC

AAACTATGACTCTTTCCTCTTTGCAGCCCTACAACTCTTCCTATCACA
```
